# First Toxicological Analysis of the Pufferfish *Sphoeroides pachygaster* Collected in Italian Waters (Strait of Sicily): Role of Citizens Science in Monitoring Toxic Marine Species

**DOI:** 10.3390/ani13111873

**Published:** 2023-06-04

**Authors:** Chiara Malloggi, Biagio Rizzo, Alice Giusti, Lisa Guardone, Laura Gasperetti, Sonia Dall’Ara, Andrea Armani

**Affiliations:** 1 FishLab, Department of Veterinary Sciences, University of Pisa, Viale delle Piagge 2, 56124 Pisa, Italy; chiaramalloggi01@gmail.com (C.M.); alice.giusti@vet.unipi.it (A.G.); lisa.guardone@izsto.it (L.G.); 2 Istituto Zooprofilattico Sperimentale del Piemonte, Liguria e Valle d’Aosta, 10154 Torino, Italy; 3 Istituto Zooprofilattico Sperimentale del Lazio e della Toscana, 00178 Roma, Italy; laura.gasperetti@izslt.it; 4 Fondazione Centro Ricerche Marine, National Reference Laboratory on Marine Biotoxins, Viale A. Vespucci 2, 47042 Cesenatico, Italy; sonia.dallara@centroricerchemarine.it

**Keywords:** marine toxins, TTX, saxitoxins, pufferfish, not indigenous species, Mediterranean, risk assessment

## Abstract

**Simple Summary:**

Human poisoning due to the unknowing consumption of toxic pufferfish could represent a risk for EU citizens, as species currently present in the Mediterranean Sea can be accidentally caught by fishermen. Thus, monitoring pufferfish in the Mediterranean area, as well as assessing their toxicity, is pivotal for proper risk management. In this study both these aspects were addressed by collecting 56 pufferfish specimens of the species *Sphoeroides pachygaster* from Italian waters (Straits of Sicily) and by analyzing samples (liver, intestine, gonads, muscle, skin) from 20 specimens for the presence of toxins. The contribution of local fishermen to specimen collection was fundamental, confirming the importance of involving citizens in the monitoring of changes in the marine environment. In fact, this is the second largest sampling of *S. pachygaster* in this area. The specimens analyzed for the presence of toxins were found to be negative. However, further studies should be conducted, especially in the light of the changing environmental scenario, which could influence the production of toxins.

**Abstract:**

Pufferfish (Tetraodontidae) inhabiting the Mediterranean Sea may represent an emerging public health risk due to the possible accumulation of marine neurotoxins such as tetrodotoxin (TTXs) and saxitoxin (STXs) in their tissues. In this study, the presence of pufferfish species in the Strait of Sicily (Lampedusa Island, Italy) was investigated using a citizen science (CS) approach, involving local fishermen. Samples (liver, intestine, gonads, muscle, skin) from 20 specimens were sent to the National Reference Laboratory on Marine Biotoxins for TTXs detection using a validated HILIC-MS/MS method on fish tissue. The presence of STXs was also screened in part of the specimens. Overall, 56 specimens identified as *Sphoeroides pachygaster* (Müller &Troschel, 1848) were collected. Data on their total length, body weight, fishing method and catch area (with relative depth temperature and salinity) were analyzed and compared with the *S. pachygaster* records reported in literature which were updated to 2022. All the analysed tissues were found to be negative for both TTXs and STXs. CS played an essential role in monitoring potentially toxic marine species in this investigation. Outcomes from this study, which is the first investigating *S. pachygaster* toxicity in Italian waters, may provide useful data for the proper assessment of this emerging risk.

## 1. Introduction

Numerous non-indigenous species (NIS), originally distributed at tropical latitudes, currently inhabit the Mediterranean Sea [1], mainly because of anthropogenic environmental modification (e.g., “Lessepsian” migration). Fish of the Tetraodontidae family (commonly known as pufferfish) are involved in this phenomenon, with at least eleven species described in the Mediterranean waters [2]. Among them, the invasive silver-cheeked toadfish, *Lagocephalus sceleratus* (Gmelin, 1789), and other species with a less invasive character, namely oceanic puffer, *Lagocephalus lagocephalus* (Linnaeus, 1758), blunthead puffer, *Sphoeroides pachygaster* (Müller and Troschel, 1848) and Guinean puffer, *Sphoeroides marmoratus* (Lowe, 1838), were reported along the Italian coasts [2]. Pufferfish represent the most common cause of tetrodotoxin (TTX) poisoning worldwide [2,3]. TTX is an umbrella term used to identify different thermostable toxins and analogues (TTXs) [4,5,6] belonging to a structurally distinct group of guanidinium alkaloids [7], and this study will utilise this nomenclature. It can be accumulated in different pufferfish tissues via the food chain [8], with concentration usually dependent on the sex, habitat, and season [4,5,6]. TTXs are mainly produced by more than 30 genera of marine bacteria [3,8]. Such TTXs-producing bacteria have been found to be associated with several aquatic organisms, including dinoflagellate [3]. In addition to pufferfish, gastropods, arthropods and cephalopods are reported as sources of poisoning [9]. 

TTXs share a high affinity with another guanidinium alkaloids neurotoxin group, saxitoxins (STXs), also known as paralytic shellfish toxins (PSTs) [7,10]. STXs are produced by marine dinoflagellates such as *Gymnodinium catenatum*, *Alexandrium* spp. and *Pyrodinium bahamense*, and by freshwater cyanobacteria such as *Cylindrospermopsis* spp. and *Lyngbya* spp. [11,12]. Given this structural affinity, TTXs and STXs share a similar mechanism of action, and the eventual ingestion of contaminated seafood leads to the blockage of Na^+^ channels [13]. Human ingestion of TTXs causes severe neurological symptoms, as voltage-dependent Na^+^ channels are blocked, preventing the conduction of these cations across the membrane. When this mechanism affects excitable cells, such as neurons and muscle cells, it leads to paralysis. If ingested in sufficient quantities, TTXs is potentially lethal [4].

Cases of TTXs/STXs human poisoning due to the unaware ingestion of several species of toxic pufferfish are reported worldwide [9,14,15]. In the Mediterranean area, human TTXs poisoning is mainly related to the unintentional consumption of *L. sceleratus* [3]. The first study investigating the toxicity of pufferfish caught in the Mediterranean Sea was performed in 2008 [16]. Since then, 17 studies on this topic have been produced (Table 1).

Among the four species recorded along the Italian coasts (*L. sceleratus*, *L. lagocephalus*, *S. pachygaster*, *S. marmoratus*), TTXs were found in all specimens of *L. sceleratus* collected from different Mediterranean areas and in some specimens of *L. lagocephalus* collected in the southwestern region, along the Tunisian coast (Table 1). In contrast, TTXs were never found in Mediterranean specimens of *S. pachygaster* (Table 1). However, poisoning cases related to the ingestion of *Sphoeroides* spp. have been reported in central–south America and in Oceania [9]. With respect to STXs, no human poisoning due to the ingestion of pufferfish is reported to date in the Mediterranean area [2], although STXs accumulation in *L. sceleratus* was described in these waters [17]. Interestingly, neither the presence of TTXs nor of STXs was investigated in pufferfish specimens collected along the Italian coasts (Table 1).

The catch area in Table 1 is categorized according Zenetos et al. [34] as follows: (i) western Mediterranean Sea (WMED); (ii) central Mediterranean Sea (CMED); (iii) Adriatic Sea (ADRIA); and (iv) eastern Mediterranean Sea (EMED). As concerns the presence of both these neurotoxins in marine organisms apart from pufferfish, TTXs were recently detected in different species of molluscs and gastropods from marine water surrounding European countries, including the UK, Greece, Netherlands, Portugal, Spain, Italy and France [3]. Some studies highlighted the presence of TTXs in bivalve mollusks collected in the Mediterranean Sea and in the Italian waters [35]. TTXs were found in in bivalve mollusks from the Northern Adriatic Sea (Italy) [36] and from the central Adriatic Sea [37]. Additionally, a high concentration of both TTXs and STXs was found in mussel from Sicily (Italy) in 2019 [38]. In this last case, the analyses were performed after the detection of algal blooms of *Alexandrium* spp. in the Ionian coasts of Sicily [38]. In this respect, the frequency of toxic blooms has increased worldwide, mainly due to global warming [39]. In fact, salinity and temperature are known to be key factors that determine the chemical toxin profile of dinoflagellates; hence, it is logical to expect toxin modifications with climate change [39].

The potential spread of TTXs and STXs, and of organisms potentially accumulating such toxins, in the Mediterranean waters represents an emerging risk for EU citizens, and unavoidably caught the attention of both the scientific community and the control authorities. Since this phenomenon is large-scale, involving citizens could help in its monitoring [40]. The so-called “Citizen Science” (CS), intended as a science where the public actively contributes to research [41], can provide information that otherwise could not be obtained, and is often applied to phenomena related to changing biodiversity [34]. Targeted campaigns and meetings have been already held to make EU citizens aware of the TTXs poisoning risk linked to the presence of pufferfish in the Mediterranean Sea [42]. A CS approach was used in our previous project entitled “Climate change and food safety: molecular, microbiological and toxicological analysis on toxic fish species in the Tyrrhenian Sea”, funded by the Italian Ministry of Health [43]. In that context, a revision of pufferfish records in the Mediterranean Sea, and specifically along the Italian coasts, was proposed for the characterization of this emerging risk in an early stage of the process [2]. Unfortunately, no specimens were collected during that project.

In this study, CS was further used through the involvement of local fishermen, with the aim of monitoring the presence of pufferfish in the Strait of Sicily (Lampedusa Island). Thanks to this, the fishermen managed to collect specimens of *S. pachygaster*, the unique pufferfish species that was captured. Considering the scarcity of data on the presence of TTXs (Table 1) in Mediterranean *S. pachygaster*, a subsample of the collected specimens was analyzed for TTXs by the Marine Research Centre (Cesenatico, Italy), the National Reference Laboratory for Marine Biotoxins (NRL). The presence of STXs was also screened in part of the specimens. By offering an in-depth view of the distribution of *S. pachygaster* in the investigated area, and by providing new toxicological data on the occurrence of TTXs (and STXs) in fish tissues, this study will contribute to a proper risk assessment in a changing environment scenario.

## 2. Materials and Methods

### 2.1. Citizen Science for the Monitoring of Pufferfish Species

#### 2.1.1. Organization of the Monitoring Activities

The first contact was established with the Competent Authority of Lampedusa (Sicily, Italy) and the Fishermen Association operating in the waters around the island in January 2020, to enquire about the fishermen’s willingness to participate in a CS project for the monitoring of pufferfish species. The willingness was confirmed by the Competent Authority, and by 20 fishermen out of a total of 40 regularly registered at the municipality. Thus, an informative brochure, including pictures and a morphological description of the 4 pufferfish species already recorded along the Italian coasts (*L. lagocephalus*, *L. sceleratus*, *S. marmoratus* and *S. pachygaster*), was produced and distributed to help fishermen with species identification (Figure S1).

#### 2.1.2. Project Dissemination

In July 2020, a meeting was organized at the Lampedusa Marine Protected Area headquarters (Sicily, Italy) by the Department of Veterinary Sciences (University of Pisa, Pisa, Italy) and the municipality of Lampedusa to present the project and to establish a collaborative relationship between interested parties. The meeting was attended by stakeholders belonging to the fishing sector of the marine area, including the Italian Institute for Environmental Protection and Research (I.S.P.R.A of Palermo), the port authorities of Lampedusa, the Fishermen Association of Lampedusa, the local administration of Lampedusa and Linosa and the local dive centers. The meeting focused on the collection of data and pufferfish specimens. Information on the pufferfish species described in the Mediterranean Sea was provided, with particular attention being paid to those already reported along the Italian coast, along with the related risks for consumers.

### 2.2. Pufferfish Specimens’ Collection and Identification

#### 2.2.1. Specimens’ Collection

The fishermen were asked to photograph and weigh each pufferfish specimen that was accidentally caught during routine fishing activities and also instructed on how to preserve (preferably frozen) and transfer them to the collection point on the island. The fishermen were also asked to record the date of each catch and additional information regarding the GPS coordinate of the approximate fishing site and the fishing method. Whole specimens (when possible) were transferred frozen from the collection point to the FishLab (Department of Veterinary Sciences, University of Pisa, Pisa, Italy). Once received, each specimen’s total length (from the apex of the snout to the caudal peduncle) was measured. Using the weight data provided by the fishermen and the length data collected by the FishLab, the overall body weight (mean and range) and the overall total length (mean and range) were calculated. Using the additional information provided by the fishermen, the depth and relative type of seabed for each GPS coordinate (corresponding to the catch point for a variable number of specimens) was evaluated with the ETOPO1 database (National Geophysical Data Centre; updated to the year 2009) [44]. In addition, the average salinity and the water temperature measured at the bottom level was assessed with the World Ocean Atlas 2018 database (Ocean Climate Laboratory of the National Oceanografic Data Centre, U.S.) [45]. Then, the mean and range of depth, salinity and temperature were calculated. The average temperature and salinity are discussed in the results section regarding TTXs detection outcomes (Section 3.3).

#### 2.2.2. Specimens’ Identification

All the collected specimens (*n* = 56, with 42 in 2020 and 14 in 2021) were morphologically identified as *S. pachygaster* using the FAO morphological keys (FishBase.org, last access 5 May 2023). The morphological identification was confirmed by molecular analyis, conducted as described in Malloggi et al. [46] on 10 specimens that were randomly selected from those received. Two distinct haplotypes of *S. pachygaster* were found. One representative *COI* sequence of each haplotype was deposited on GenBank (Accession number OR002130-31).

### 2.3. Collection of Records of S. pachygaster in the Mediterranean and Italian Waters

All the results obtained from the previous sections were compared to the available literature on the Mediterranean records of *S. pachygaster*. To do this, the *S. pachygaster* records reported in the study of Guardone et al. [2] (from 1979 until 2016) were updated until 2022 by searching and selecting new records using the same criteria [2]. The same data considered by Guardone et al. [2] were collected from the new records (Table 2). The following parameters were calculated for the overall dataset (from 1979 to 2022): (1) the overall number of recorded specimens (also classified per date of catch), (2) the total specimen length (mean and range calculated as in Section 2.2.1), (3) the specimen body weight (mean and range calculated as in Section 2.2.1), (4) the depth of the catch area (mean and range) of the collected specimens. The number of specimens used for the calculation of these parameters was different due to the high variability in the data presentation observed in the literature records.

### 2.4. TTXs Detection

A subsample of 20 specimens were selected for TTXs analysis among the 42 collected in 2020 (as the sampling was completed that year, from February to September). All specimens heavier than the mean body weight calculated by Guardone et al. [2], corresponding to 1075.3 g, were used, as TTXs presence is often associated with larger sizes [17]. Since, in 2020, only 7 large specimens (>1075.3 g) were collected, the other 13 smaller specimens were included to reach at least 20 specimens for use in TTXs detection. The dates of catch of these 13 selected smaller specimens were distributed throughout the entire year, to ensure that at least one specimen was analyzed per month. Tissue from the 5 organs (liver, gonads, skin, muscle and intestine) in which usually TTXs concentrate [15,57] was sampled, obtaining a total of 100 samples. All the tissue samples were identified by a univocal specimen code, packaged separately, and sent to the NRL for TTXs detection. TTXs extraction and quantification were carried out using hydrophilic interaction liquid chromatography coupled with tandem mass spectrometry (HILIC-MS/MS) [58,59]. This method, which allows for the identification and quantification of TTXs and PSTs in bivalve mollusks, was also validated on fish matrix by the Italian NRL. Table S1 reports the UPLC gradient conditions, the HILIC-MS/MS set of parameters employed and transitions for TTXs identification in accordance with the method based on electrospray ionization-mass spectrometry (ESI-MS), respectively. An at least six-point calibration curve ranging from 0.2 µg/L to 400 µg/L was used (R2 ≥ 0.98) for the quantification of the toxin. The limit of detection (LOD) was 3 µg/kg and the limit of quantification (LOQ) 10 µg/kg. TAB1 UPLC LC gradient conditions were used (11 min runtime). The concentration was calculated on the basis of extrapolation from the linear regression line (R2 ≥ 0.98) by applying the following formula:

$$\mathrm{TTX}\left(\mu g/\mathrm{kg}\right)=\frac{{\mathrm{conc}}_{\mu \mathrm{moli}/l}\times {\;\mathrm{PM}}_{\mathrm{TTX}}\times {\;V}_{\mathrm{fin}}\times {\;\mathrm{dil}}_{\mathrm{SPE}\;}\times {\;\mathrm{dil}}_{\mathrm{inj}}}{\mathrm{gr}}$$

where: $conc_{µmoli/l}$ = concentration of extract TTXs; $PM_{TTX}=$ molecular weight of TTX (319.27 gr/mol); $V_{fin}=final\;extraction\;volum$; $dil_{SPE\;}=sample\;dilution\;in\;the\;SPE\;step$; $dil_{inj}$; gr = sample dilution for injection: grams of extracted sample.

## 3. Results and Discussion

### 3.1. Citizen Science for Monitoring the Presence of Pufferfish Species in the Strait of the Sicily (Lampedusa Island)

CS is an increasingly popular approach for monitoring and scientific research [60]. It possesses several advantages, including a cost-effective way of gathering data, especially over a large spatial and temporal area, and data of a fine and spatial temporal resolution. However, data from CS projects can vary in quality, especially when protocols are too complex or demanding, or recording needs to be repeated over time or in different localities. In this respect, the CS is often most effective when the approach is simple [60].

To date, there have been thousands of EU projects using a CS approach, especially in the areas of life and the natural sciences [61,62]. In 2015 it was estimated that 25% of the projects involving CS worldwide concerned marine or coastal areas [63]. In fact, CS may allow for biodiversity to be monitored at a large-scale and for the detection of long-term changes in the ecosystem [64]. CS could have high potential for monitoring the distribution, behavior, and dynamics of NIS, and make a difference to early detection, allowing for large geographical areas to be covered at a lower coast. Indeed, the presence of invasive species and their impact on the marine ecosystem remain largely unrecorded due to logistical difficulties and the need to invest huge resources [65]. Encarnação et al. [66] highlighted that, in terms of records, the contribution of CS to the marine NIS monitoring was almost double than terrestrial NIS.

According to over 8500 records of fish and mollusks NIS, mollusks were reported to occur in the European marine waters from 1905 to 2019 thanks to CS [64]. It is interesting to underline that the highest number of fish records related to *L. sceleratus* [66]. This species shows an unprecedented invasive character in terms of both abundance and geographical range [67], and could be considered among the fastest expanding Lessepsian immigrants.

In the Mediterranean basin, such projects have been carried out to monitor coral reefs, gelatinous plankton, fish and NIS [65]. In our previous project [43], the dissemination activities were carried out using brochures, posters, reports, socials and newspaper articles. In addition, direct contact was established with fishermen, divers and control authorities to create a network for the collection of data for a better assessment of the risk related to the spreading of toxic pufferfish. The project did not result in the collection of pufferfish specimens.

In the CS project ‘‘AlienFish’’, aimed at monitoring rare fish NIS in Italian waters, the pivotal role of sea users and professional fishermen was highlighted. Therefore, in this study, we decided to directly involve fishermen to monitor the presence of pufferfish in the area of interest. Interestingly, when the informative brochure describing the four species recorded along the Italian coasts (see Section 2.1.1) was distributed, some fishermen immediately recognized the species *S. pachygaster*. The other pufferfish species were unknown. Aware of the difficulties that the general public can encounter when dealing with scientific research, we structured our CS project to be as simple as possible, focusing on specimen collection as the main objective, and selecting a limited fishing area.. Specimen collection began immediately after the first contact in January 2020, demonstrating how aware the fishermen were of the regular presence of *S. pachygaster* in the water surrounding the Island. In fact, during the informal preliminary contact with fishermen, we were informed that this species is commonly fished as a by-catch during routine fishing. The most plausible hypothesis for *S. pachygaster*’s expansion in the Mediterranean Sea is its entrance from the Atlantic Ocean (from the West African coastline) through the Strait of Gibraltar [68]. However, a Lessepsian migration cannot be excluded given the circumglobal distribution of the species [2].

**Figure 1:**
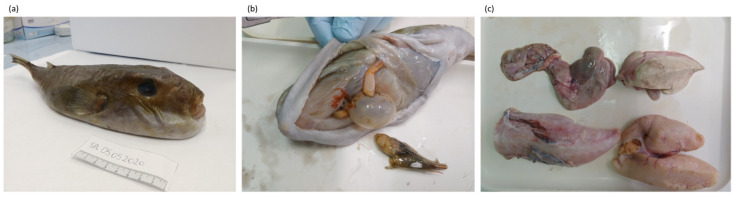
One of the whole specimens collected in this study (**a**) with relative internal organs (**b**,**c**).

The 56 specimens analyzed in this study were collected by only five fishermen out of the twenty who confirmed their willingness to participate in this project (Table 3). Despite this low participation rate (25%), we were able to collect a large number of specimens and a the most part of specimens were in a good state of preservation when received (example in Figure 1). In fact, our study ranks second, after that by Ragonese et al. [69], dated over two decades ago, in which 403 specimens of *S. pachygaster* were collected in the Strait of Sicily (Italy). In both cases, the collection was achieved thanks to the involvement of local fishermen. In addition to collecting a large number of specimens, we also obtained substantial data linked to the specimens, although a few cases of data loss occurred; for instance, not all the specimens were weighed on board (see Section 3.2.2).

### 3.2. Occurrence of S. pachygaster in the Mediterranean and Italian Waters: Data from Our Specimen Collection and the Literature

#### 3.2.1. Overall Specimens’ Number and Date of Catch

According to the review by Guardone et al. [2], 716 specimens of *S. pachygaster* were recorded in the Mediterranean Sea from 1979 to July 2016. By analyzing the 13 new records (Table 2), we observed that the overall number of Mediterranean *S. pachygaster* was 45 (from 2017 to 2022). By adding the 56 specimens collected in the present study to those reported from the literature ([2] and new records), 817 specimens were recorded in the Mediterranean waters from 1979 to 2022. Interestingly, 561 of them (68.7%) were caught in the central Mediterranean (CMED) and 550 (98% of the CMED specimens) were caught in the Strait of Sicily (Italy).

Regarding the 56 specimens collected in this study, 42 (75%) were caught in 2020 (February–September) and 14 (25%) in 2021 (January–May) (Table 3). The mean number of specimens collected per capture (*n* = 16) was 3.3, with a range of 1–5 specimens for all considered months, except for July 2020, in which 12 specimens were collected in a single capture. These data cannot be compared with those from the literature because only the catch ranges were given in many cases (e.g., “collected from 2016 to 2020”). However, extrapolating the number of specimens found in the new records for the period 2020–2021 (corresponding to the collection period of this study), we found that five specimens were collected in 2020 and only one in 2021 (Table 2). Only the one from 2021 was recorded in the same catch area considered in this study (Strait of Messina, Italy). Thus, this study reports the first 2020 records for *S. pachygaster* in Sicily.

#### 3.2.2. Specimens’ Total Length and Body Weight

Although specific instructions were provided to the fishermen, not all the specimens were weighed on-board. In addition, considering the difficulty of logistics, some specimens were not stored in the correct way, limiting the measurements. In total, we were able to obtain both weight and length for 45 out of 56 specimens (80.3%). All the specimens from 2020 were weighed and measured, while this was possible for only three specimens from 2021. Therefore, data related to weight and length were only complete for 2020. The project was intended to last only one year (2020); thus, the instruction given to fishermen were not always followed during the captures in 2021 and only two out of the five fishermen continued to collect specimens. The total length and body weight of each specimen are reported in Table 3. The mean total length was 254 mm (range of 160–450 mm), while the mean body weight was 635.1 g (range weight of 100–2570 g). By analyzing the same data from new literature records, we observed that the mean total specimen length was 407.4 mm (*n* = 5), with a range of 190–520 mm (*n* = 17), while the mean body weight was 1022.2 g (*n* = 9), with a range of 130–2420 g (*n* = 24). The mean total length, with relative ranges, found in the collected specimens and in new records was in line with those reported in Guardone et al. [2]. The mean body weight of the specimens collected in our study was lower than that of Guardone et al. [2] (despite being calculated for a similar number of specimens: *n* = 45 and *n* = 41, respectively), while the mean total specimen length of the update was higher but calculated on a very low number of subjects (*n* = 5).

#### 3.2.3. Additional Information on Catch Areas and Fishing Method

Overall, 16 GPS coordinates corresponding to the fishing areas were provided by fisherman (Figure S2). Since all the specimens were caught using trawls, each GPS coordinate represents an indicative point of the route travelled during the day’s outing. Fishermen normally sound the bottom of small areas of sea during the day. These 16 GPS coordinates can be grouped into 6 distinct areas (from A to F) (Figure S2) in which captures were concentrated. Each area is associated with from one to six GPS coordinates, corresponding to the number of captures (Figure S2). Details on fishermen, captures and specimens collected per area are given in Table S2.

The area of competence of the Lampedusa fishermen, characterized by the highly irregular bottom bathymetry where very great depths are reached [70], represents the ideal habitat for *S. pachygaster*. This species is benthopelagic, usually living between 50 and 250 m [71], but can also live up to 480 m depth [72]. In this study, the specimen mean catch depth, calculated using the 16 GPS coordinates, was 96.8 m, with a range of 16–170 m (Table S3). However, most part specimens (*n* = 33; 58.9%) were caught at depths over 120 m. The mean depth of specimen catches observed in the new records was higher (181 m), with a range from 10 to 400 m (Table 2). However, without considering the record from Hussein et al. [52] regarding the catch of one specimen from the coast, the range varies from 120 to 400. Overall, the comparison of the analysis of the specimen depth of catches from this study and that from the new records is in line with the results of Akbora et al. [28], who reported that this species is mostly fished at depths greater than 100 m, and consistent with what was reported in the study of Guardone et al. [2] (mean depth 149.3 m).

Regarding the type of seabed, specimens were caught in medium sand or sand (*n* = 7; 43.8%) and fine sand or silty sand (*n* = 9; 56.2%) in deeper waters (Table S3). The mean depth of catch calculated for the two types of seabed was 54.7 m and 129.4 m, respectively. In the new records, only one reported the type of seabed (soft bottom), without specifying the depth (Table 2).

Because of its natural habits, *S. pachygaster* is normally a by-catch of semi-industrial fisheries as purse seines bottom trawls, gillnets, trammel-nets, bottom long-lines and drift longline [52]. According to some authors, this species could be considered invasive because it could constitute an economic burden for fishermen, who have to discard a large number of specimens from their gear [73]. Indeed, the Strait of Sicily was selected by Ragonese et al. [69] because of the presence of a large trawling fleet. Bottom trawling is still the most significant fishing activity in this area [74] and, as previously mentioned, the fishing method most frequently involved in the catch of *S. pachygaster*. These fishing methods are in line with the seabed types found in this study and with those reported by Guardone et al. [2] (muddy or muddy/sandy). In fact, trawls can only be used with a sandy seabed. Regarding the 45 specimens described in the new records, 44 (97.8%) were caught, while only 1 specimen was found stranded (Table 2). Among those caught, the most-used fishing method, reported for 28 (63.6%) specimens, was trawling (*n* = 22; 78.6%), followed by longline (*n* = 3; 10.7%), and trammel-net (*n* = 2; 7.1%), and only one specimen was caught by a fisherman at 300 m from the coast (*n*= 1; 3.6%) (Table 2).

Overall, the results of this study support literature, confirming that, since the 1990s, the area of the Strait of Sicily has hosted an established population. Some authors have suggested that this species arrived here prior to the date of the first Mediterranean record [71]. Moreover, the wide size range and the numerical consistent found in this study, as in that of Ragonese et al. [69], indicates that a complete sexual cycle exists, supporting the fact that the species can be considered stable in these waters. Therefore, *S. pachygaster* could be considered an established or naturalized population according to the definitions reported in Zenetos et al. [75].

### 3.3. TTXs Detection Outcomes

In the European regulationsno limit is set for TTXs pufferfish: all the species belonging to Tetraodontidae, Molidae, Diodontidae and Canthigasteridae families should not be commercialized according to Regulation (EC) No. 853/2004. However, the same Reg. sets a maximum PSTs concentration of 800 µg STX eq./kg in bivalve mollusks, echinoderms, tunicates and marine gastropods [76]. This is also the maximum level in the US and Canada, while in Japan the limit is higher (2 mg STX eq./kg) [35,77]. The EFSA Panel on Contaminants in the Food Chain recognized the need for further data on the acute oral toxicity of TTX [78]. In addition, the EFSA panel suggested that, since TTXs and STXs have similar modes of action and their toxicity is probably cumulative, they could perhaps be combined to yield one health-based guideline value [78]. For this reason, it would be accurate to evaluate them together.

In this study, for the first time, 20 specimens of *S. pachygaster* collected along the Italian coasts were analyzed for the presence of TTXs (Table 3). In addition, 10 specimens (among those analyzed for TTXs) (Table 3) were randomly screened for STXs. In fact, although the method used in this study was not fully validated for the presence or absence of STXs in fish tissue, as previously mentioned, EFSA recommends assessing the presence of both these guanidinium neurotoxins [78]. In addition, Mediterranean *S. pachygaster* was never investigated for the presence of STXs, although poisoning cases associated with specimens belonging to the genus *Sphoerodies* were reported in North America; however, these were rather dated [17,18]. All the 20 specimens analyzed for TTXs, of which 7 (35%) were above the mean body weight reported in Guardone et al. [2], were found to be negative. In fact, TTXs concentrations were found to be below the LOQ in all analyzed tissue samples (<10 µg/kg). Also the ten specimens that were randomly analyzed (three large and seven small) for STX detection were found to be negative.

Data regarding the toxicity of *S. pachygaster* are discordant. It is considered non-toxic along the Japanese coast [79,80,81], and Amano et al. [82] reported no TTX in specimens caught in the East China Sea in 2009. Interestingly, *S. pachygaster* was the only species negative for TTX among the five analyzed in that study [82]. It was also reported as non-toxic in the Mediterranean [83]. However, *S. pachygaster* in the pacific waters was considered weakly toxic by Noguchi and Arakawa [8].

Pufferfish saxitoxin and tetrodotoxin-binding protein (PSTBP) is one of the most intriguing proteins related to the toxicity of pufferfishes. If PSTBP plays an important role in the transport and accumulation of TTX, the diverse toxicity among pufferfish species would be attributed to functional and/or copy number changes in PSTBPs and its ancestral proteins, TBT-bps [84]. Genes for homologous TTXs and STXs binding proteins were also detected in *S. pachygaster* [84]. In a study by Nagashima et al. [85], liver tissue slices of non- toxic species of pufferfish incubated with TTX accumulated the toxin after 8 h, regardless of the toxicity of the species. In addition, poisoning cases related to the ingestion of *Sphoerides* spp. (including *S. testudineus* and *S. maculatus*) are reported in Centre–South America and Oceania [9], suggesting that species belonging to this genus can accumulate toxins. Furthermore, TTXs was found in two *Sphoeroides* species (*S. annulatus* and *S. lobatus*) caught near the Mexican Peninsula of Baja California [86]. Although human poisonings due to pufferfish ingestion are usually recollected to TTXs, we should consider that, in the large majority of cases, no laboratory analysis is carried out to confirm the involved toxin, and that TTX intoxication is clinically diagnosed on the basis of anamnesis and of the symptoms [9]. In fact, the occurrence of TTXs in some pufferfish species has been reported together with STXs [15]. For example, STX-positive (not TTX) pufferfish belonging to the genus *Sphoeroides* have been recorded since 2002 in the USA [87]. Worldwide, STXs has been found along with TTXs in several marine and freshwater pufferfish species in areas of the Far East, including Japan, Philippines and Thailand [15]. Several species belonging to the *Sphoeroides* genus were recently found to contain STXs in American waters [15,88]. Therefore, it would be more accurate to say that *S. pachygaster* could accumulate TTXs and STXs under particular conditions [53]. Only three studies have investigated the presence of TTXs in specimens of *S. pachygaster* caught in the Mediterranean basin [18,24,33], and no one has investigated STXs in this species (Table 1). Reverté et al. [18] and Rambla-Alegrè et al. [24] analyzed *S. pachygaster* specimens caught along the Mediterranean coast of Spain, and all were found to not contain TTXs. Recently, Ulman et al. [33] reported the absence of TTXs in one *S. pachygaster* specimen caught in the Eastern Mediterranean Sea (EMED). To date, the exact biosynthetic pathways and genes implicated in TTX production are still unclear [89]. However, the available information suggests that marine organisms, such as pufferfish, acquire TTXs from other toxic organisms bearing bioconcentrated TTXs through the food chain, starting with marine bacteria [8]. Some studies have demonstrated that wild toxic pufferfish species bred under controlled conditions and artificially fed with toxin-free diets do not present toxins, while non-toxic pufferfish become toxic when fed TTXs-containing diets [8]. A possible explanation for this could be that marine organisms consume microalgae (dinoflagellates), which host TTXs-producing symbiotic bacteria, and accumulate them along the trophic chain [3]. This theory, involving microalgae in bacterial symbiosis, is supported by the fact that significant concentrations of TTXs were detected in dinoflagellate cell cultures [90]. Another study in which TTXs was found in bivalve mollusks collected in the Gulf of Syracuse (Sicily, Italy) supports this hypothesis. In fact, the highest abundance of TTXs in dinoflagellates was found at the same time as toxins were found in mussels. A possible correlation between the presence of TTXs in mollusks and dinoflagellates was also hypothesized in a study conducted in the Aegean Sea (Greece) [91]. This demonstrates how phytoplankton could play a key role in TTXs transfer [38]. Whereas TTXs in pufferfish is mostly of bacterial origin, STXs originates from microalgae, principally from dinoflagellates in marine species and from cyanobacteria in freshwater species [11]. If pufferfish consume crustaceans and mollusks exposed to dinoflagellate blooms, they may become vectors for STXs [15]. In fact, it has been assumed that Japanese Takifugu found to have a high concentration had eaten bivalves exposed to the toxic dinoflagellate [92]. However, questions about the origin of STXs arise from the study of Pinto et al. [57], in which, although pufferfish were found to be positive for the toxin, STXs were never found in marine organisms from Madeira Island, and STXs-producing dinoflagellates, such as *Gymnodinium catenatum* and *Alexandrium* spp., are not known in this area [93]. However, some xanthid crabs, horseshoe crabs and marine snails may possess STXs not associated with dinoflagellates. For instance, some authors reported that pufferfish and bivalves could acquire TTXs by consuming toxic planoceri flatworms [37]. In addition, because the skin and mucus of all toxic pufferfish species contains higher levels of STXs than other tissue, it could be suggested that, in the absence of toxic food items, STXs could be taken up via an undetermined pathway. Despite this, the main source of TTXs and STXs remains bivalve mollusks [94]. As previously mentioned, TTXs production in dinoflagellate species may be due to its relationship with symbiotic bacteria, which are able to produce toxic compounds when the environmental factors are favorable. Water temperature, salinity and depth are among the environmental factors that play a key role in the occurrence of TTXs in marine organisms. In this study, the water mean temperature and salinity at point of catch was 15.8 °C (range 15.3–17.6 °C) and 36.8 °C (range 36–38 °C), respectively (temperature and salinity, along with GPS coordinates, are reported in Table S3). According to the available literature, TTXs were typically present in shellfish collected at temperatures of 15 °C [89], although Turner et al. [59] did not correlate TTXs occurrence and water temperature. Bordin et al. [36] reported TTXs in shellfish collected in the Northern Adriatic Sea (Italy) in May, when the water temperature ranged between 19°C and 21°C, while Vlamis et al. [91] found TTX-positive mussels during the same period, but they did not report the water temperature. In the study of Leão et al. [95], a low concentration of TTXs in shellfish from Spain was found in samples collected in July from an intertidal area with a water temperature of 17 °C. Abiotic factors could stimulate TTXs production in bacteria such as *Vibrio* [59], and bacterial counts seemed to be influenced by temperature and salinity. Higher counts occurred in warmer months and in water with lower salinity values [96]. As regards depth, shellfish positive for TTXs were collected in inter-tidal or water where the salinity level was lower than open marine waters [89]. Moreover, Dell’Aversano et al. [38] highlighted how warm water, a low water depth and strong solar radiation are potential critical factors for TTXs accumulation. In addition to TTXs contamination with a level up to 6.4 mg TTX eq/Kg, they found a significant level of contamination from PST in mussels collected in Syracuse Bay (Sicily) during spring–summer of 2015, 2016 and 2017, demonstrating that TTXs levels can also be associated with STXs levels in mussels. Bivalve mollusks were collected at 10 sites during a mixed bloom of *Alexandrium* spp., known to be a producer of STXs [38]. In the study of Bacchiocchi et al. [37], bivalve mollusks with the highest toxin concentrations came from shallow waters (20–40 cm) with high temperatures (20–40 °C) and salinity (from 34.3 to 36), suggesting that environmental factors such as strong solar radiation and a relatively high water temperature may favor TTXs accumulation in mussels.

All these factors could explain the occurrence of TTXs and STXs in bivalve mollusks or pufferfish collected from different geographical areas. In fact, other studies reported the co-occurrence of TTXs and STXs in bivalves and gastropods [38,97,98]. Therefore, the different toxicity patterns of *S. pachygaster* and *L. sceleratus* could be related to their habitat depths, although both feed on mollusks, shrimps and small teleosts. *S. pachygaster* usually lives at over 100 m depth, while *L. sceleratus* is mostly found in shallow waters (mean 24.3 m) [2]. Interestingly, TTXs were not reported in the Mediterranean Sea until 2008 [16]. It is likely that the propagation of TTXs-producing bacteria in the Mediterranean waters was favored by climatic change and TTXs carrier species being transported through the Suez Canal [16]. Therefore, we cannot exclude the possibility that other non-toxic Mediterranean species, such as *S. pachygaster*, may eventually accumulate TTXs through the trophic chain.

## 4. Conclusions

This is the first survey to evaluate the toxicity of *S. pachygaster* specimens collected along the Italian coasts (Strait of Sicily). With 56 specimens collected in 2020–2021, this sampling is the largest, with the exception of one performed over 15 years ago. The specimens’ collection was possible thanks to the involvement of local fishermen, who were trained in the identification of pufferfish species inhabiting the Italian coasts, confirming the key role of the CS in the monitoring of marine NIS. All the analyzed specimens were found to be negative for the presence of TTXs, and parts of them screened for STXs were also found to be negative. However, despite the non-toxicity of *S. pachygaster* in the study area at present, the issue should be monitored, especially in light of climate change, which affects water temperature and could influence the production of toxins by bacteria and dinoflagellates. Overall, this study shows that *S. pachygaster* is currently well established in the Strait of Sicily and provides new data that are essential for the proper assessment of this emerging risk.

## Figures and Tables

**Table 1:** Available studies analysing the presence of TTXs in different pufferfish species collected in the Mediterranean Sea (only the original studies related to the listed specimens are reported). The number of analysed specimens, the catch area and the results of the TTXs analysis are reported.

Specie	N° of Specimens	Catch Area	TTX Results	Reference
*L. lagocephalus*	10	WMED–Tunisia, Gabes region	Positive	Saoudi et al. [16]
*L. sceleratus*	6	EMED–Aegean Sea	Positive	Katikou et al. [17]
*S. pachygaster*	1	WMED–Spain	Negative	Revertè et al. [18]
*L. sceleratus*	1	EMED–Greece	Positive	
*L sceleratus*	1	EMED–Aegean Sea	Positive	Bane et al. [19]
*L. sceleratus*	5	EMED–Turkey	Positive	Kırımer et al. [20]
*L. sceleratus*	16	EMED–Mersin Bay	Positive	Kosker et al. [21]
*L. sceleratus*	20	EMED–Turkey	Positive	Acar et al. [22]
*L. sceleratus*	20	EMED–Egypt	Positive	Alabssawy [23]
*L. lagocephalus*	14	WMED-–Spain	Negative	Rambla-Alegre et al. [24]
*L. sceleratus*	1		Positive	
*S. pachygaster*	5		Negative	
*Torquigener flavimaculosus*	16	EMED–Mersin Bay	Positive	Kosker et al. [25]
*L. sceleratus*	2	EMED–Aegean Sea	Positive	Leonardo et al. [26]
*L. sceleratus*	40	EMED–Mersin Bay	Positive	Kosker et al. [27]
*Lagocephalus spadiceus*	40	EMED–Mersin Bay	Negative	
*Lagocephalus suezensis*	40	EMED–Mersin Bay	Positive	
*L. sceleratus*	16	EMED–Cyprus	Positive	Akbora et al. [28]
*L. sceleratus*	1	ADRIA	Positive	Ujević et al. [29]
*L. sceleratus*	83	EMED–Crete	Positive	Christidis et al. [30]
*L. sceleratus*	112	EMED–Egypt	Positive	Farrag et al. [31]
*L. sceleratus*	3	EMED–Lebanon	Positive	Hassoun et al. [32]
*L. guentheri*	1	EMED	Negative	Ulman et al. [33]
*L. suezensis*	1		Positive	
*S. pachygaster*	1		Negative	

**Table 2:** New records of *Sphoeroides pachygaster* collected in the Mediterranean Sea with relative analyzed data. The catch is categorised as in Table 1.

Date of Catch	Catch Area	N of Specimens	Reference	Type of Record	Fishing Method	Total Lenght (mm)	Body Weight (g)	Depth (m)	Type of Seabed
2012–2015	CMED–Strait of Sicily	7	Cammilleri et al. [47]	Caught	Trawl	-	130–1750	-	-
2014–2016	WMED–Spain	5	Rambla-Alegre et al. [24]	Caught	-	-	-	-	-
2015–2016	CMED–Ionian Sea	5	Carlucci et al. [48]	Caught	Trawl	-	-	-	-
2016–2017	EMED–Egypt	4	Ragheb et al. [49]	Caught	Trawl	-	-	-	-
NR	CMED–Malta	9	Vella et al. [50]	Caught	-	266–465	385–2275	-	-
April 2017	EMEDLebanon	3	Gerovasileiou et al. [51]	Caught	Long line	344–417	-	-	Soft
2020	WMED–Algerian coast	2	Hussein et al. [52]	Caught	Fisherman Trawl	360.6 409.5	2250 2420	10 120	-
March 2020	EMED–Northen Cyprus	1	Akbora et al. [53]	Caught	Trammel- net	520	1200	250	-
May 2020	EMED–Turkey	1	Erguden et al. (reprted in [54])	Caught	-	490	-	400	-
July 2020	EMED–Turkey	1	Kizilkaya and Akyol [54]	Caught	Trammel-net	257	-	125	-
August 2021	CMED–Strait of Messina	1	[55]	stranded	-	-	-	-	-
March 2022	EMED–Turkey	5	Karakuş et al. [56]	caught	Trawl	190–410	10–1405	140–240	-
NR	EMED–Turkey	1	Ulman et al. [33]	caught	-	-	-	-	-

**Table 3:** Number of collected specimens divided per catch date (and relative fisherman—Fm) with relative body weight (gr) and total length (cm), size (S = small; L = large) and type of analysis performed (TTXs/STXs).

Catch Date (Fishermam)	N of Specimens	Specimen Code	Body Weight (gr)	Total Lenght (cm)	Size	Toxin Analysis
18/02/20 (Fm1)	1	P01	196	175	S	TTXs
29/02/20 (Fm2)	2	P02(A)	199	220	S	TTXs STXs
		P02(B)	215	190	S	-
13/03/20 (Fm1)	3	P03(A)	670	270	S	TTXs
		P03(B)	122	190	S	TTXs STXs
		P03(C)	100	160	S	-
11/04/20 (Fm3)	3	P04(A)	687	260	S	TTXs STXs
		P04(B)	170	117	S	-
		P04(C)	160	131	S	-
5/05/20 (Fm2)	2	P05(A)	743	260	S	TTXs
		P05(B)	264	220	S	TTXs STXs
21/05/20 (Fm1)	3	P06(A)	2570	420	L	TTXs
		P06(B)	136	190	S	-
		P06(C)	202	210	S	-
21/05/20 (Fm4)	1	P07(A)	841	360	S	TTXs STXs
29/05/20 (Fm2)	2	P08(A)	1726	430	L	TTXs
		P08(B)	394	250	S	TTXs STXs
4/06/20 (Fm5)	3	P09(A)	207	210	S	TTXs
		P09(B)	179	190	S	-
		P09(C)	169	200	S	-
1/07/20 (Fm5)	12	P10(A)	349	220	S	TTXs STXs
		P10(B)	199	210	S	-
		P10(C)	163	190	S	-
		P10(D)	296	235	S	-
		P10(E)	202	200	S	-
		P10(F)	378	250	S	-
		P10(G)	574	260	S	-
		P10(H)	290	220	S	-
		P10(I)	205	210	S	-
		P10(J)	296	220	S	-
		P10(K)	523	270	S	-
		P10(L)	496	270	S	-
4/08/20 (Fm4)	3	P11(A)	1655	360	L	TTXs
		P11(B)	523	270	S	TTXs STXs
		P11(C)	496	270	S	-
20/08/20 (Fm5)	3	P12(A)	2145	445	L	TTXs STXs
		P12(B)	271	220	S	TTXs STXs
		P12(C)	203	200	S	-
2/09/20 (Fm4)	4	P13(A)	264	230	S	-
		P13(B)	2280	450	L	TTXs STXs
		P13(C)	1850	450	L	TTXs STXs
		P13(D)	1640	420	L	TTXs STXs
30/01/21 (Fm5)	5	P14(A)	ND	ND	-	-
		P14(B)	ND	ND	-	-
		P14(C)	ND	ND	-	-
		P14(D)	ND	ND	-	-
		P14(E)	ND	ND	-	-
8/02/21 (Fm5)	5	P15(A)	860	360	S	-
		P15(B)	ND	ND	-	-
		P15(C)	ND	ND	-	-
		P15(D)	ND	ND	-	-
		P15(E)	ND	ND	-	-
14/04/21 (Fm5)	2	P16(A)	ND	ND	-	-
		P16(B)	ND	ND	-	-
14/05/21 (Fm1)	2	P17(A)	1148	410	L	-
		P17(B)	1407	430	L	-

## Data Availability

Data are included in the manuscript and available in Supplementary Tables S1–S3 attached to the text; further data are also available from the authors upon request.

## Supplementary Materials

The following supporting information can be downloaded at: https://www.mdpi.com/article/10.3390/ani13111873/s1, Figure S1: informative brochure distributed to the fishermen; including pictures and morphological description of the four pufferfish species already recorded along the Italian coasts (*Lagocephalus lagocephalus*, *Lagocephalus scerelatus*, *Sphoeroides marmoratus* and *Sphoeroides pachygaster*); Figure S2: GPS coordinates of specimen captures with relative areas (A, B, C, D, E, F) in which captures were concentrated and fishermen. Table S1: HILIC-MS/MS set of employed parameters; Table S2: Details of captures, fishermen (Fm) and specimens collected per area; Table S3: Fisherman (Fm), catch date, number of specimens caught, GPS coordinates with relative depth, type of seabed, temperature and salinity.
