# Supplementary material for: First Toxicological Analysis of the Pufferfish Sphoeroides pachygaster Collected in Italian Waters (Strait of Sicily): Role of Citizens Science in Monitoring Toxic Marine Species
Source: Animals (Basel). 2023 Jun 4;13(11):1873. doi: 10.3390/ani13111873 (PMC10252100; doi:10.3390/ani13111873)
Supplement: Supplementary file 1 [file animals-13-01873-s001.zip › animals-2409952-supplementary.pdf]

Figure S1

Opuscolo informativo

- Materiale illustrativo a disposizione degli operatori del mare contenente indicazioni per il riconoscimento visivo delle specie ittiche invasive con particolare attenzione alle specie potenzialmente tossiche per la salute umana.

FISH LAB

DIPARTIMENTO DI SCIENZE VETERINARIE - UNIVERSITÀ DI PISA

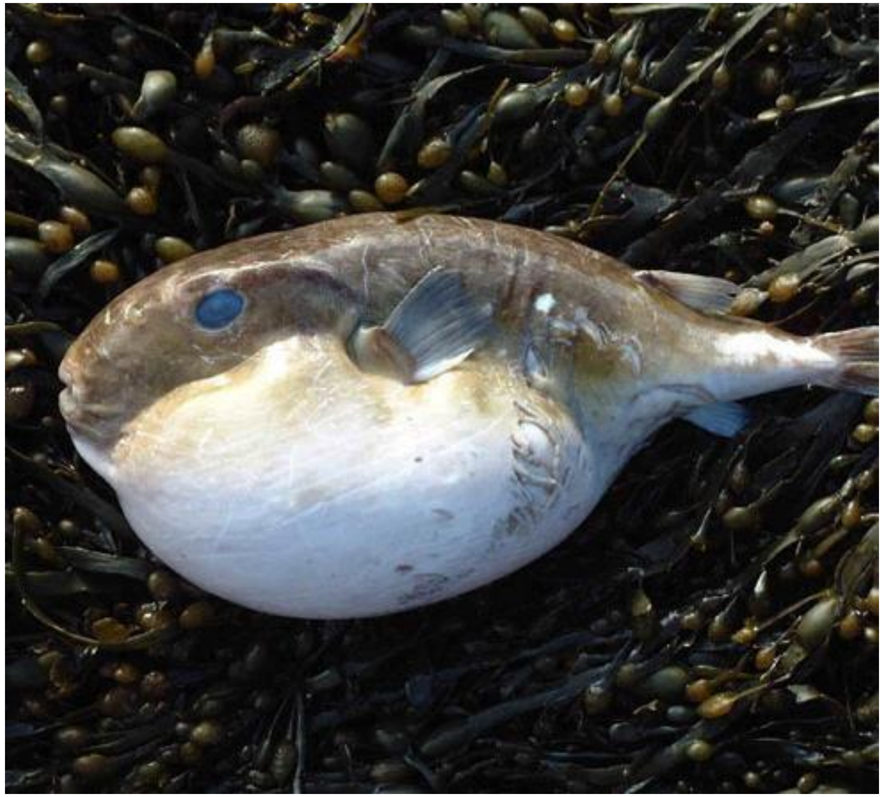

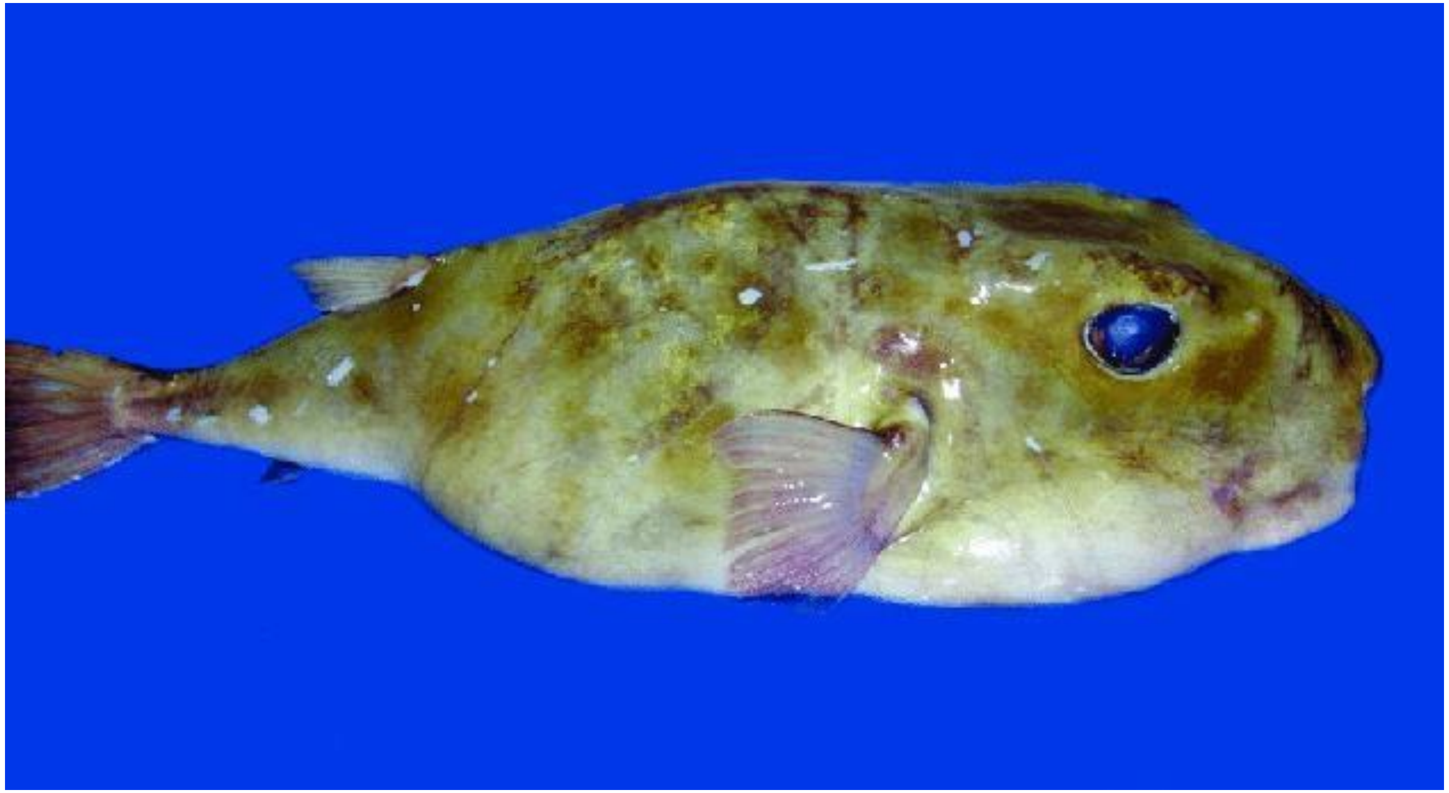

Sphoeroides pachygaster

- Ha il caratteristico aspetto dei pesci palla, con corpo tozzo, gonfiabile, pinne dorsale ed anale brevi ed arretrate, testa ed occhi grandi, bocca dotata di 4 grossi denti. La pinna caudale ha margine dritto. le pinne ventrali sono assenti mentre le pinne pettorali sono ampie.
- Il colore è bruno sul dorso ed argenteo su fianchi e ventre.
- Raggiunge i 25 cm di lunghezza, le femmine sono più grandi dei maschi.

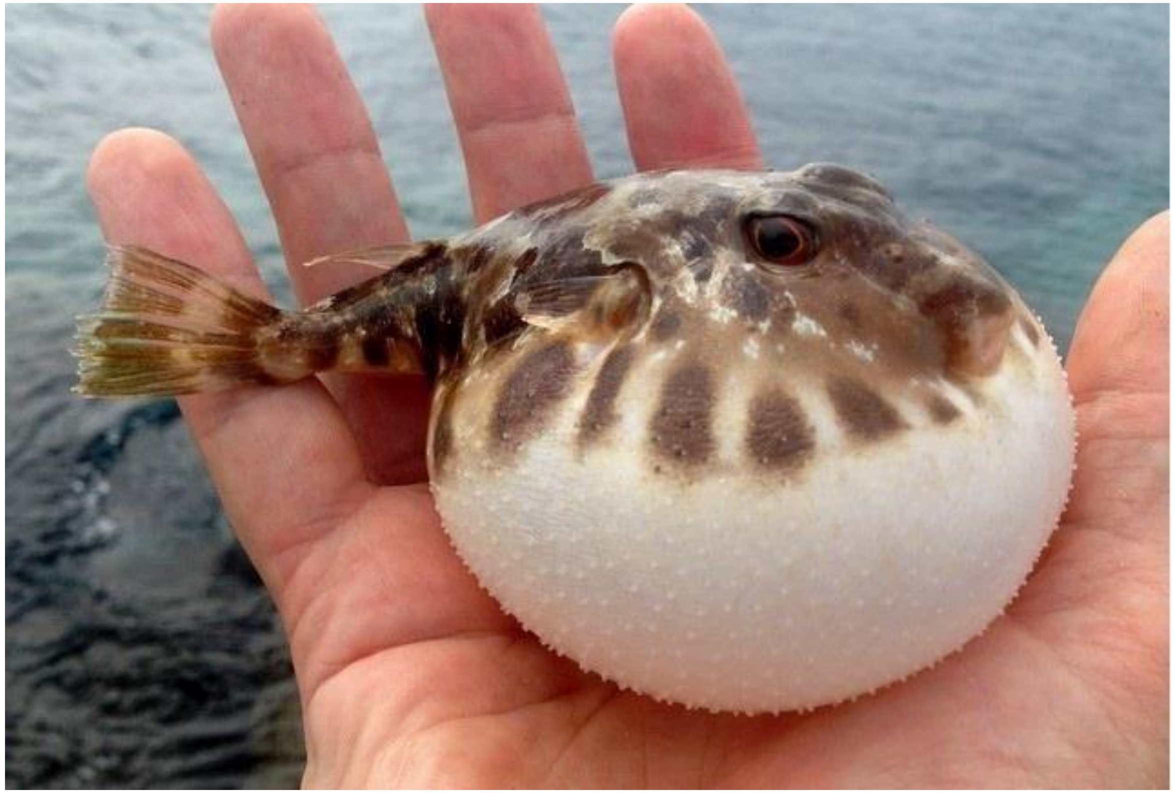

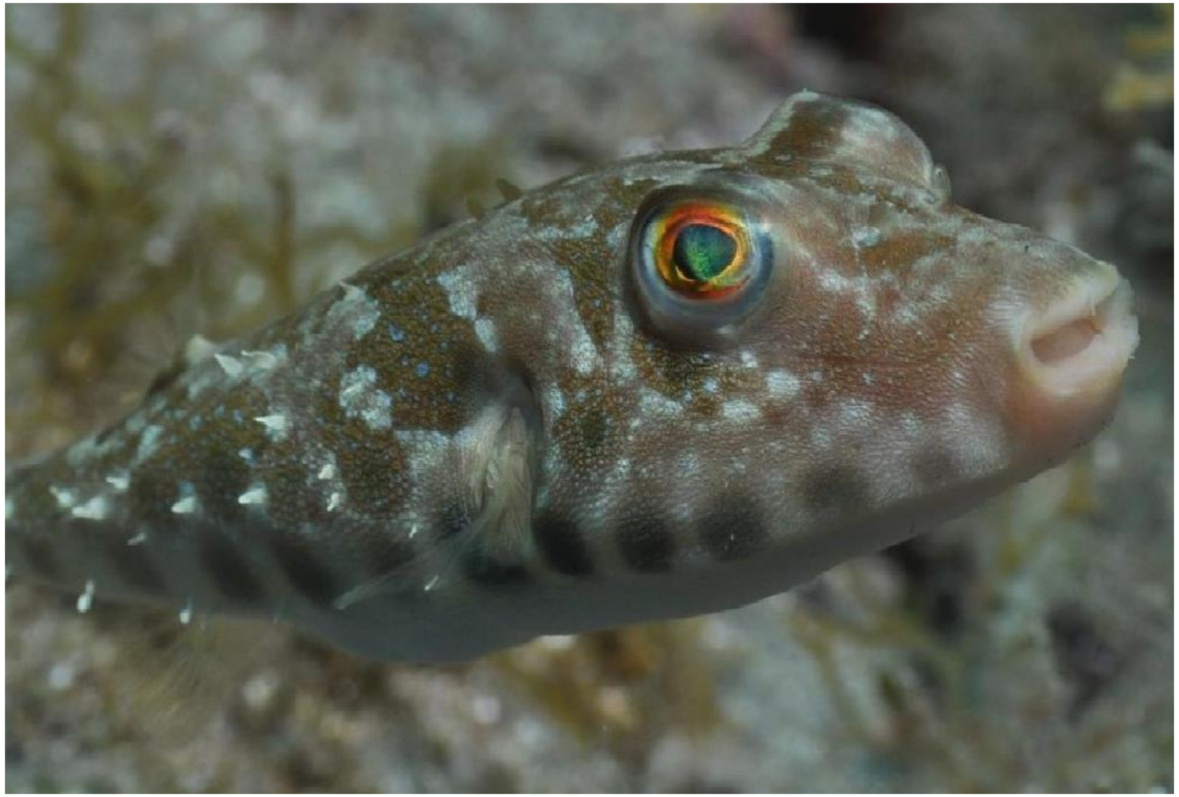

Sphoeroides marmoratus

- Corpo gonfiabile. Narici all'estremità di un'appendice ovale. Pinna caudale arrotondata, dorsale, anale e pettorali biancastre quasi trasparenti
- Il corpo si presenta di colore verde-grigio marroncino, con ventre bianco e provvisto di spine. Sul dorso sono presenti una serie di macchie scure ed un paio di lembi neri sulla superficie medio-dorsale del corpo.
- Pesce di piccola taglia.

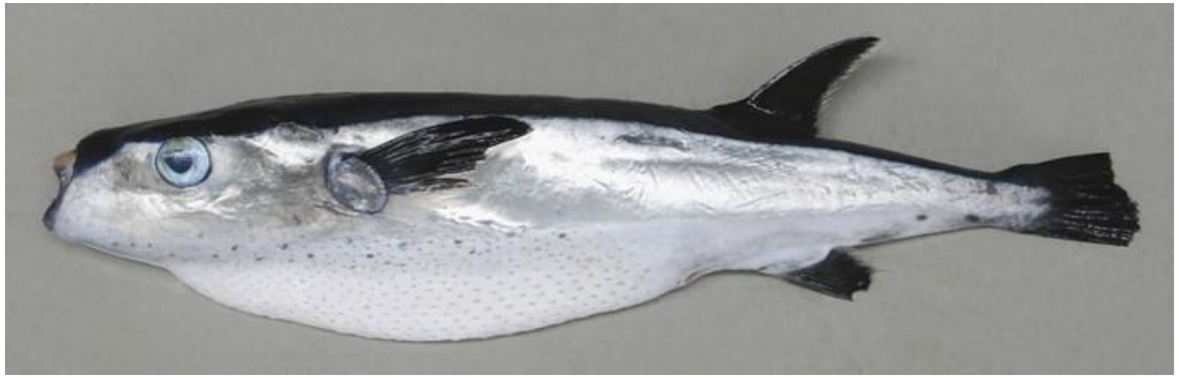

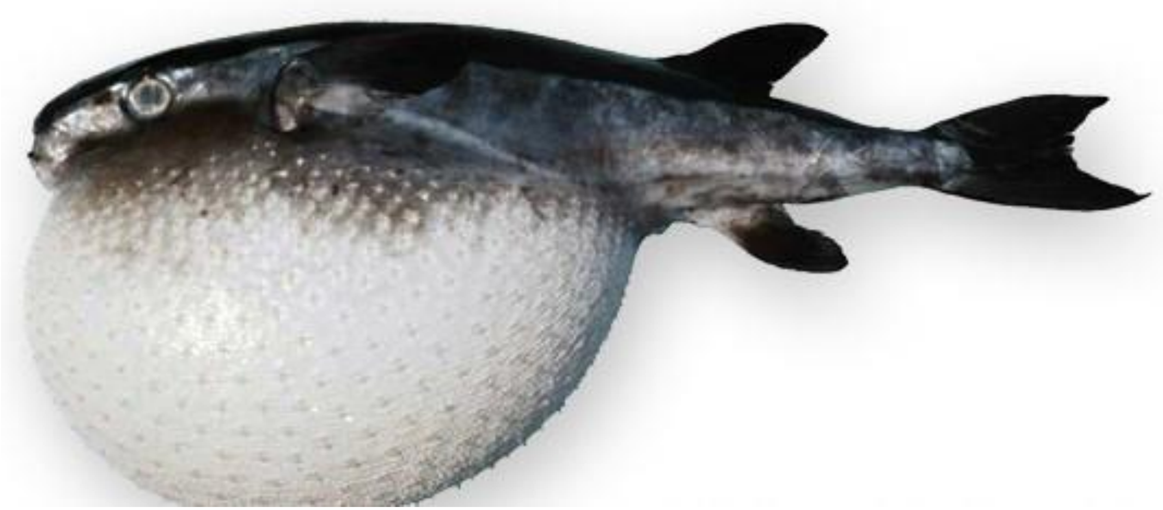

Lagocephalus lagocephalus

- La livrea è caratteristica ed è il miglior criterio per distinguerlo da specie affini, il dorso è infatti di colore blu senza macchie, i fianchi argentei presentano due bande laterali ed il ventre è bianco.
- Le pinne pettorali dorsali ed anali sono scure posizionate lontane e posteriormente.
- Il corpo, nel complesso, può raggiungere i 65 cm, peso massimo registrato 3,2 kg

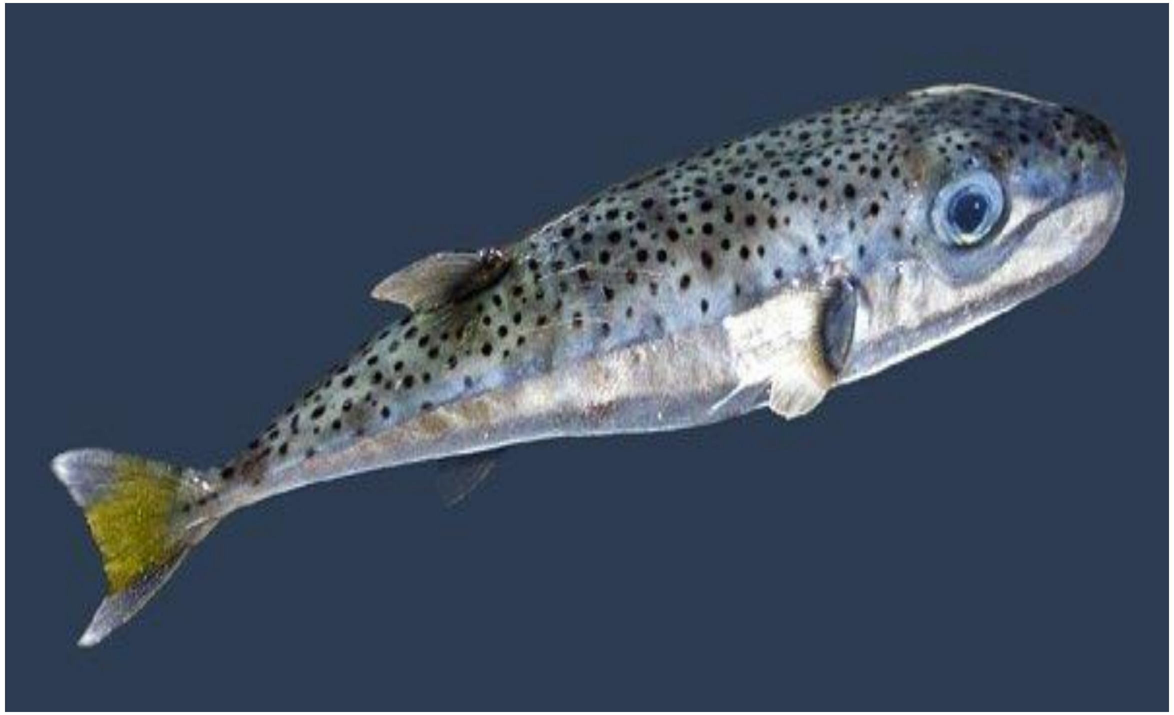

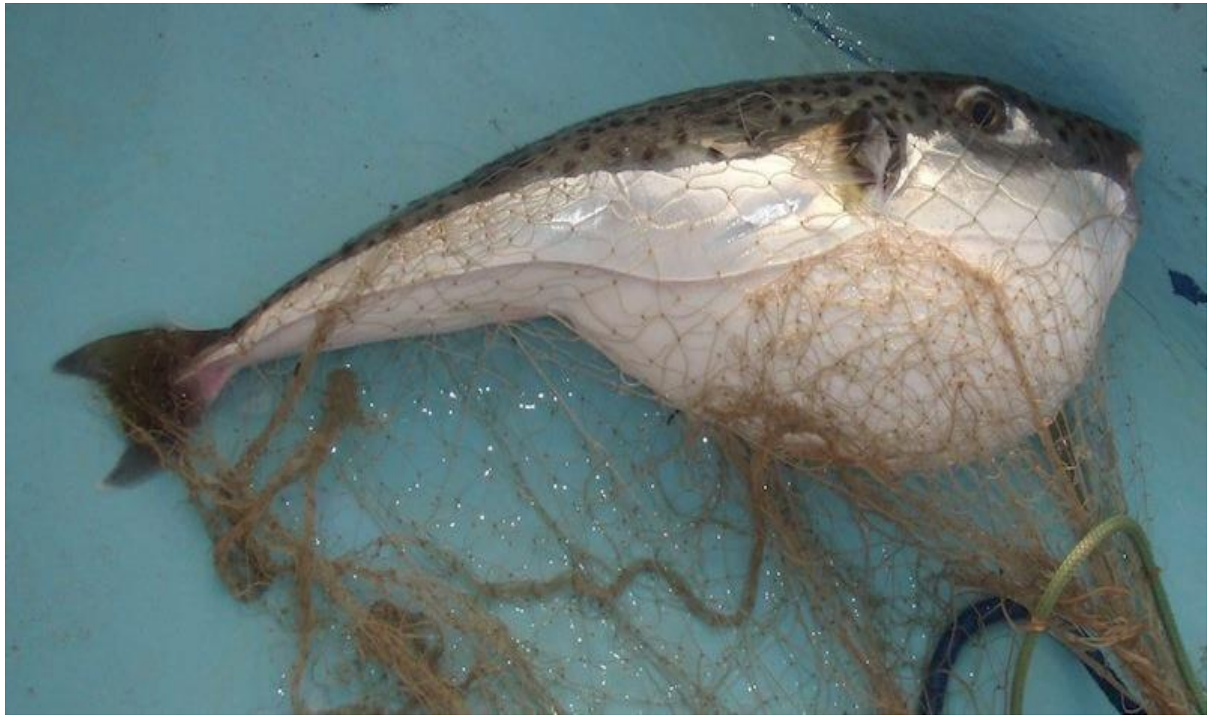

Lagocephalus sceleratus

- Il pesce palla maculato è il più tossico ed invasivo dei pesci palla .
- Può raggiungere 110 cm di lunghezza e 7 kg di peso.
- Ha un corpo allungato e compresso ai lati. Non presenta squame, ma possiede delle piccole spinule sul ventre e sulla superficie dorsale. Le pinne dorsale e anale si trovano in posizione posteriore.
- Il dorso è verdastro con macchie regolari marrone scuro o nere, lungo i fianchi dalla bocca alla pinna caudale è ben visibile una banda argentea; il ventre è bianco. È infine evidente una macchia argentea davanti all’occhio.

[illegible]

dell’operatore del mare, come specie invasiva/aliena/tropicale.

- Il Dipartimento di Scienze Veterinarie dell’università di Pisa in collaborazione con il comune di Lampedusa e Linosa ringraziano tutti coloro che prenderanno parte alle attività di monitoraggio delle specie ittiche invasive potenzialmente tossiche per la salute umana al fine di contribuire, fornendo informazioni essenziali , all’analisi dell’evoluzione faunistica del Mar Mediterraneo a seguito dei cambiamenti climatici.

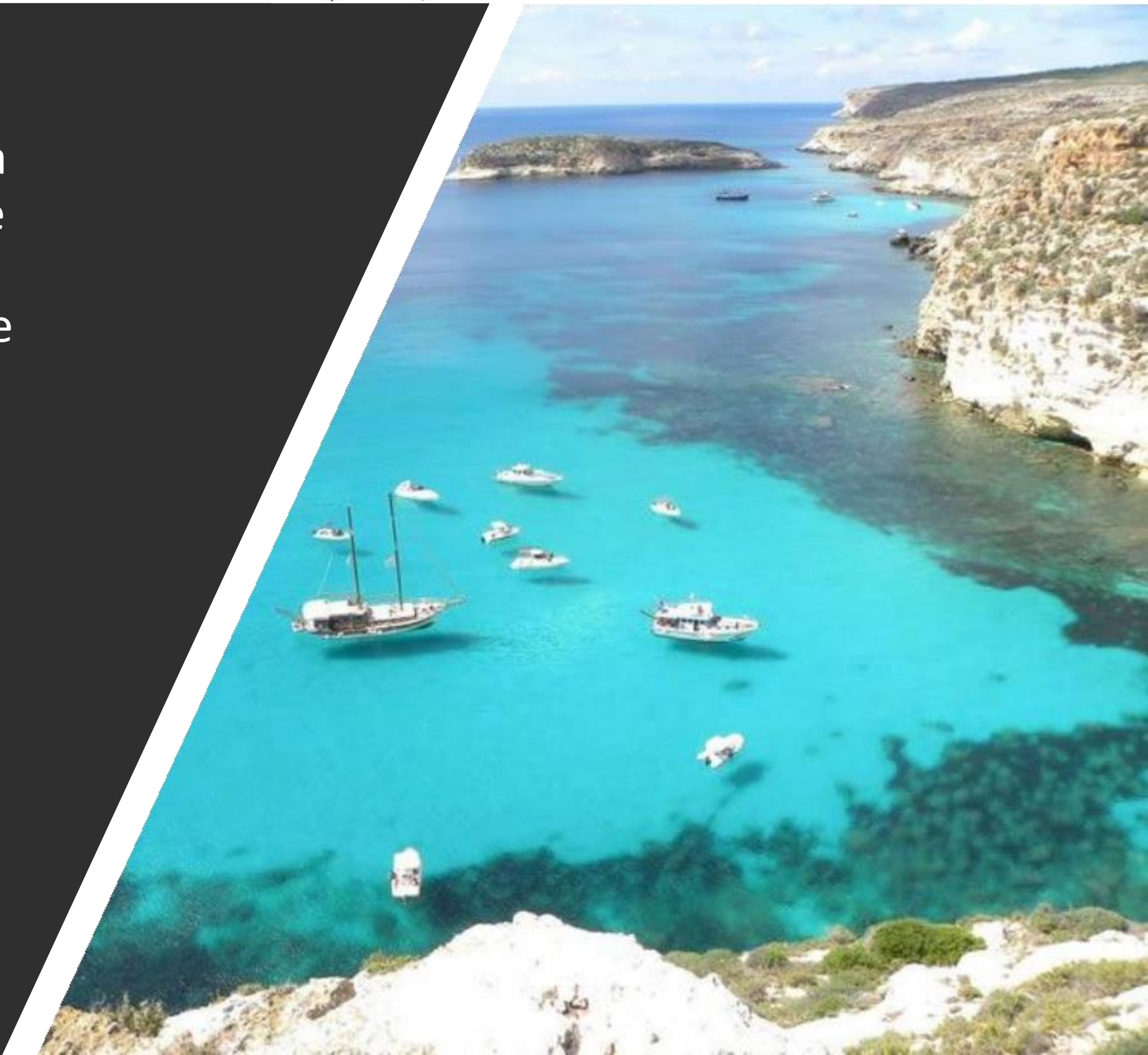

Figure S2. GPS coordinates of specimens captures with relative areas (A, B, C, D, E, F) in which captures were concentrated and fishermen.

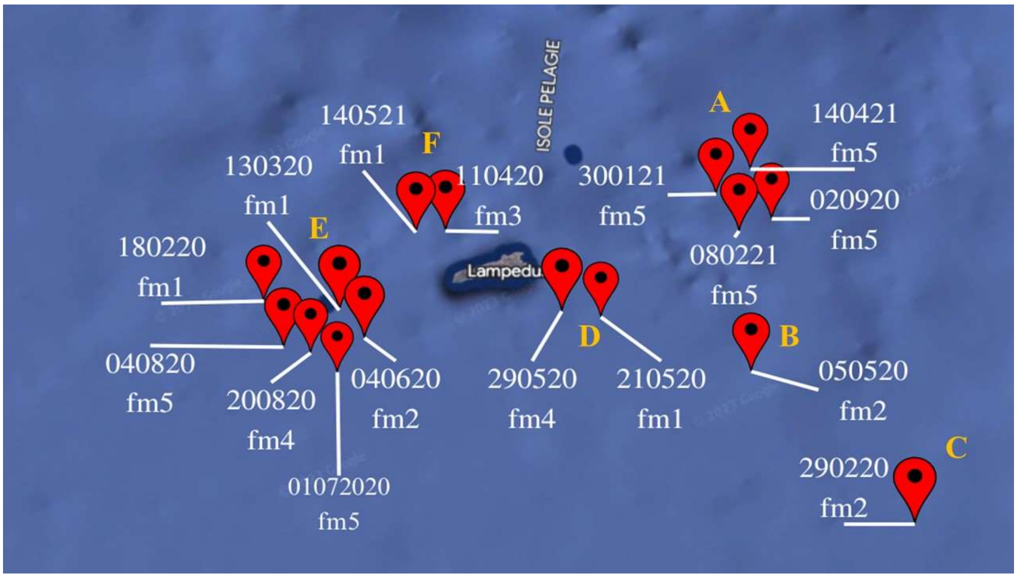

Table S1. HILIC-MS/MS set of employed parameters

1) UPLC LC gradient condition (run time: 11 min)

Time	Flow Rate (mL/min)	% A1	% B1	Max. Pressure Limit (bar)
Initial	0.400	2.0	98.0	1200.00
4.00	0.400	2.0	98.0	1200.00
7.50	0.400	50.0	50.0	1200.00
9.00	0.500	50.0	50.0	1200.00
9.50	0.500	5.0	98.0	1200.00
10.0	0.800	2.0	98.0	1200.00
10.60	0.800	2.0	98.0	1200.00
10.61	0.400	2.0	98.0	1200.00
11.00	0.400	2.0	98.0	1200.00

2) XEVO TQ-XS CONDITIONS

Parameter	Positive Ionisation ESI+
Capillary Voltage (kV)	3.0
Cone voltage (V)	20
Source temperature (°C)	150
Desolvation Temperature (°C)	500
Cone Gas Flow (L/Hr)	150
Desolvation Gas Flow (L/Hr)	1000
Collision gas flow (ml/Min)	0.15
Nebuliser Gas flow (Bar)	7

3) Transition XEVO TQ XS MS/MS (MRM)

Analogue	Precursor Ion	Product Ion	Collision Energy	Cone	Polarity
TTX	320,1	302,1	25	40	Positive
TTX	320,1	162,1	38	40	Positive

Table S2. Details of captures, fishermen (Fm) and specimens collected per area are reported.

Areas	N° of capture	Date of catch	N° of specimens collected per capture	Fishermen	Total N° of specimens per area
**A**	4	02/09/2020	4	Fm5	16
		30/01/21	5	Fm5	
		08/02/21	5	Fm5	
		14/04/21	2	Fm5	
**B**	1	05/05/20	2	Fm2	2
**C**	1	29/02/20	2	Fm2	2
**D**	2	21/05/20	4	Fm1	6
		29/05/20	2	Fm4	
**E**	6	18/02/20	1	Fm1	25
		13/03/20	3	Fm1	
		04/06/20	3	Fm2	
		01/07/20	12	Fm5	
		04/08/20	3	Fm5	
		20/08/20	3	Fm4	
**F**	2	11/04/20	3	Fm3	5
		14/05/21	2	Fm1	

Table S3. Fisherman (Fm), catch date, number of specimens caught, GPS coordinates with relative depth, type of seabed, temperature and salinity.

Fisherman	Catch date	N° of specimens	GPS coordinate	Depth (m)	Type of seabed	T bottom level (°C)	Salinity (‰)
Fm1	18/02/20	1	lat 35° 34′ 8,35″ N/long 12° 15′ 45,8″ E	69	Medium sand or sand	15	37.7
Fm2	29/02/20	2	lat 35° 00′ 9,30″ N/long 13°04′ 21,0″ E	83	Fine sand or silty sand	15.6	37.9
Fm1	13/03/20	3	lat 35° 32′ 9,84″ N/long 12° 21′ 47,2″ E	78	Medium sand or sand	15	38.1
Fm3	11/04/20	3	lat 35° 35′ 50,0″ N/long 12° 32′ 00″ E	82	Fine sand or silty sand	15	38
Fm2	05/05/20	2	lat 35° 14′ 54,0″ N/long 12° 49′ 39,0″ E	16	Medium sand	16.5	37.7
Fm1	21/05/20	4	lat 35° 21′ 9,06″ N/long12° 42′ 7,87″ E	48	Medium sand or sand	16.6	37.7
Fm4	29/05/20	2	lat35° 26′ 38,0″ N/long 12° 40′ 36,0″ E	31	Medium sand or sand	17.6	38
Fm2	04/06/20	3	lat 35° 30′8,60″ N/long 12° 21′ 31,0″ E	69	Medium sand or sand	15.9	37.8
Fm5	01/07/20	12	lat 35° 29′ 8,75″ N/ long 12° 58′ 8,50″	121	Fine sand or silty sand	15.8	38.2
Fm5	04/08/20	3	lat 35° 29′ 8,77″ N/ long 12°58′ 8,00″ E	122	Fine sand or silty sand	15.6	36
Fm4	20/08/20	3	lat 35° 27′ 4,00″ N/ long 12°10′ 38,0″ E	72	Medium sand or sand	16.3	36
Fm5	02/09/20	4	lat 35° 29′ 8,00″ N/long 12° 58′ 8,00″ E	122	Fine sand or silty sand	15.8	38.1
Fm5	30/01/21	5	lat 35° 29′ 7,50″ N/long 12°57′6,10″ E	139	Fine sand or silty sand	15.5	38.5

Fm5	08/02/21	5	lat 35° 28′ 50,0″ N/long 12° 56′ 40,0″ E	170	Fine sand or silty sand	15.3	38.6
Fm5	14/04/21	2	lat 35° 28′ 36,5″ N/long 12° 56′ 44,8″	160	Fine sand or silty sand	15.3	38.6
Fm1	14/05/21	2	lat 35° 42′ 58,3″ N/ long 12° 29′ 18,6″ E	166	Fine sand or silty sand	15.3	38.7
